# Inter-rater agreement between humans and computer in quantitative assessment of computed tomography after cardiac arrest

**DOI:** 10.3389/fneur.2022.990208

**Published:** 2022-10-13

**Authors:** Martin Kenda, Zhuo Cheng, Christopher Guettler, Christian Storm, Christoph J. Ploner, Christoph Leithner, Michael Scheel

**Affiliations:** ^1^Department of Neurology With Experimental Neurology, Freie Universität Berlin and Humboldt-Universität zu Berlin, Charité—Universitätsmedizin Berlin, Berlin, Germany; ^2^BIH Charité Junior Digital Clinician Scientist Program, Berlin Institute of Health at Charité—Universitätsmedizin Berlin, BIH Biomedical Innovation Academy, Berlin, Germany; ^3^Department of Neuroradiology, Freie Universität Berlin and Humboldt-Universität zu Berlin, Charité—Universitätsmedizin Berlin, Berlin, Germany; ^4^Department of Nephrology and Intensive Care Medicine—Circulatory Arrest Center Berlin, Corporate Member of Freie Universität Berlin and Humboldt-Universität zu Berlin, Charité—Universitätsmedizin Berlin, Berlin, Germany

**Keywords:** cardiac arrest (CA), neuroprognostication, computed tomography, automated image analysis, resuscitation, inter-rater agreement, brain imaging

## Abstract

**Background:**

Head computed tomography (CT) is used to predict neurological outcome after cardiac arrest (CA). The current reference standard includes quantitative image analysis by a neuroradiologist to determine the Gray-White-Matter Ratio (GWR) which is calculated *via* the manual measurement of radiodensity in different brain regions. Recently, automated analysis methods have been introduced. There is limited data on the Inter-rater agreement of both methods.

**Methods:**

Three blinded human raters (neuroradiologist, neurologist, student) with different levels of clinical experience retrospectively assessed the Gray-White-Matter Ratio (GWR) in head CTs of 95 CA patients. GWR was also quantified by a recently published computer algorithm that uses coregistration with standardized brain spaces to identify regions of interest (ROIs). We calculated intraclass correlation (ICC) for inter-rater agreement between human and computer raters as well as area under the curve (AUC) and sensitivity/specificity for poor outcome prognostication.

**Results:**

Inter-rater agreement on GWR was very good (ICC 0.82–0.84) between all three human raters across different levels of expertise and between the computer algorithm and neuroradiologist (ICC 0.83; 95% CI 0.78–0.88). Despite high overall agreement, we observed considerable, clinically relevant deviations of GWR measurements (up to 0.24) in individual patients. In our cohort, at a GWR threshold of 1.10, this did not lead to any false poor neurological outcome prediction.

**Conclusion:**

Human and computer raters demonstrated high overall agreement in GWR determination in head CTs after CA. The clinically relevant deviations of GWR measurement in individual patients underscore the necessity of additional qualitative evaluation and integration of head CT findings into a multimodal approach to prognostication of neurological outcome after CA.

## Introduction

Hypoxic-ischemic encephalopathy (HIE) remains a major cause of death and disability following cardiac arrest (1). Many CA survivors remain with disabling neurological symptoms ranging from cognitive and movement disorders to severe impairments of consciousness (2). Reliable neurological outcome prediction after CA is a challenging task. When deciding on continuation or withdrawal of life sustaining therapies, an accurate prognosis is crucial. Currently, a combination of several prognostic investigations is recommended: repeated neurological examination, electroencephalography (EEG), somatosensory evoked potentials (SSEP), serum neuron-specific enolase (NSE) and brain imaging (3).

Despite efforts for standardization, there is heterogeneity in prognostic performance between raters and centers for most of the tests used. Inter-rater agreement on EEG interpretation is fair to substantial (4) and good on SSEP interpretation for the prediction of poor outcome in comatose patients early after CA (5).

Head CTs are obtained early after CA to rule out intracranial causes for the arrest and later after CA (typically within 3–5 days) to assess the degree of HIE. The current reference standard for assessing head CT imaging is interpretation by a neuroradiologist (“qualitative analysis”). Typical characteristics of HIE include global brain edema frequently visible as sulcal effacement, loss of discrimination between gray and white matter, pseudo-subarachnoid hemorrhage or hypodense cortex and gray matter basal ganglia structures (“reversal sign”) (6). International guidelines recommend using imaging findings of “diffuse and extensive anoxic injury” for prediction of poor neurological outcome without providing a clear definition of this finding (3, 7, 8). Studies on the inter-rater reliability of qualitative assessment of head CT have consequently found poor to moderate agreement on the presence and/or severity of HIE between different raters, sites, and specialties (9, 10).

Quantitative analysis could aid in reducing Inter-rater variability. Determination of the gray-white matter ratio (GWR) by manual placement of small regions of interest (ROI) in different gray and white matter target regions has been evaluated in many mostly retrospective, single center studies (11, 12). Protocols for GWR determination are not fully standardized and differ relevantly between centers (13). Our group recently developed and successfully tested an algorithm for automated GWR assessment that uses linear and non-linear co-registration with MRI-based standard atlases to determine ROIs (14).

Limited data is available on the Inter-rater agreement in GWR determination, especially with respect to different levels of expertise. Moreover, there are no studies that investigate the agreement between conventional human raters and automated assessment by a computer algorithm.

Therefore, this study aims at the following:

1. To assess inter-rater agreement of GWR assessment between 3 human raters with different levels of expertise.
2. To assess the agreement between human raters and automated computer GWR assessment.
3. To identify potential sources of Inter-rater variability.
4. To evaluate the impact of Inter-rater variability on prognostic performance.

## Methods

### Patients

The study was approved by the local ethics committee. We retrospectively included 353 patients from a previously published cohort of our CA database from the circulatory arrest center of a large academic hospital (15). One hundred eleven patients received native head CTs within the first 7 days after CA. Three patients had technically compromised data files and could not be reanalyzed. Thirteen patients with additional cerebral pathologies such as older ischemic lesions, hemorrhage or severe motion artifacts in imaging were excluded (Supplementary Figure 1). Patients were treated with targeted temperature management (TTM, 33°C for 24 h) according to the guidelines (16). Clinicians were blinded to the results of quantitative CT analysis but had access to the radiologic report. Neurological outcome was assessed by the treating physicians at hospital discharge using the cerebral performance category (CPC) scale and dichotomized in “good” (CPC 1–3) and “poor” outcome (CPC 4–5) for statistical analysis (17).

### GWR determination

All raters were blinded to clinical information except for the overall context of CA and blinded to each other's results. Images were rated by a board-certified neuroradiologist with 13 years of clinical expertise, a resident neurologist with 3 years of experience in post-cardiac arrest care and a final year medical student who underwent a short, 3-h training session to assess GWR beforehand but had no clinical experience in the field. Using Horos (Version 3.1.2, The Horos Project, https://horosproject.org), each rater was asked to bilaterally place 10 mm^2^ ROIs into the putamen and posterior limb of the internal capsule (PLIC).

For automated analysis, we used a previously published algorithm, modified to use circular ROIs instead of atlas maps (14). In summary, CTs are first co-registered in a linear and non-linear mode to a standardized CT template in an MRI-based standard space using the *FNIRT* and *FLIRT* functions from FSL (Version 5.0.9, Analysis Group, FMRIB, Oxford, United Kingdom) (18). The reverse transformation fields are then used to automatically place predefined standardized 10 mm^2^ circular ROIs into the center of target regions in the individual CT spaces. Mean hounsfield units (HU) in the ROIs were measured and GWR was calculated as $\frac{HU\;Putamen}{HU\;PLIC}$.

### Statistical analyses

For statistical analysis, we used RStudio (Version 1.4.1653, RStudio, Boston, MA) with the *pROC*-package (19) for receiver operating characteristics (ROC), the *ggpubr*-package for data visualization and the *psych*-package (20) for ICC calculation. Intraclass correlation (ICC) (ICC 3, two-way mixed, single measure model) was conducted to measure overall and pairwise Inter-rater agreement on the radiodensity (HU) and GWR between the reference standard neuroradiologist and the other three raters. We also calculated ICC (ICC3k, Two-way mixed, average measure) between a mean of three human raters and the computer.

Intraclass correlation values < 0.2 were considered as *poor* agreement, between 0.21 and 0.4 as *fair* agreement, between 0.41 and 0.6 as *moderate* agreement, 0.61 and 0.80 as *good* agreement, and values > 0.80 as *very good* agreement (21). For comparison between the raters, we used the Wilcoxon test for the rating results and the DeLong's test for the AUCs. “Severe HIE”, was assumed if GWR was <1.10 (22). Statistical significance was defined as p<0.05. Confidence intervals (*CIs*) for neurological outcome prediction were calculated with 95% CIs using the Wilson score method.

## Results

### Patients

Ninety five patients with native head CTs were eligible for analysis. In the predominantly male (66.3%) study cohort (Table 1), the majority had an OHCA (83.2%) and a non-shockable rhythm (69.5%). Half of the patients had a primary cardiac cause of arrest, the other half either a respiratory (35%) or other cause (intoxication, metabolic, and unknown). Median length of ICU stay was 9 days [IQR 4–23]. At ICU discharge, 56 (59%) patients had a poor neurological outcome (CPC 4–5), 9 of which in unresponsive wakefulness syndrome (UWS; CPC 4), the other 47 dead (CPC 5). CTs were acquired at a median of 4 h (IQR, 1–19.5) after cardiac arrest.

**Table 1:** Baseline data of 95 CA patients.

**Parameter**	**Overall**
*n*	*95*
Age (years)	61 [48–73]
**Sex**	
Male	63 (66.3%)
Out-of-hospital Cardiac Arrest	79 (83.2%)
Shockable Rhythm	29 (30.5%)
**Primary cause of arrest**	
Cardiac	47 (49.5%)
Respiratory	35 (37.2%)
Other	13 (13.6%)
Time to ROSC (min)	16 [12–24]
Total Adrenalin Dose (mg)	2 [1–5]
APACHE Score	30 [23–35]
Length of ICU stay (days)	9 [4–23]
Time on Ventilator (hours)	204 [108–512]
CT acquisition (hours after CA)	4 [1–19.5]
**Neurological Outcome at ICU Discharge**	
CPC 1	23 (24.2%)
CPC 2	14 (14.7%)
CPC 3	2 (2.1%)
CPC 4	9 (9.5%)
CPC 5	47 (49.5%)

Median [IQR] for continuous and numbers (%) for categorical data.

### Inter-rater agreement in GWR determination

**Figure 1:**
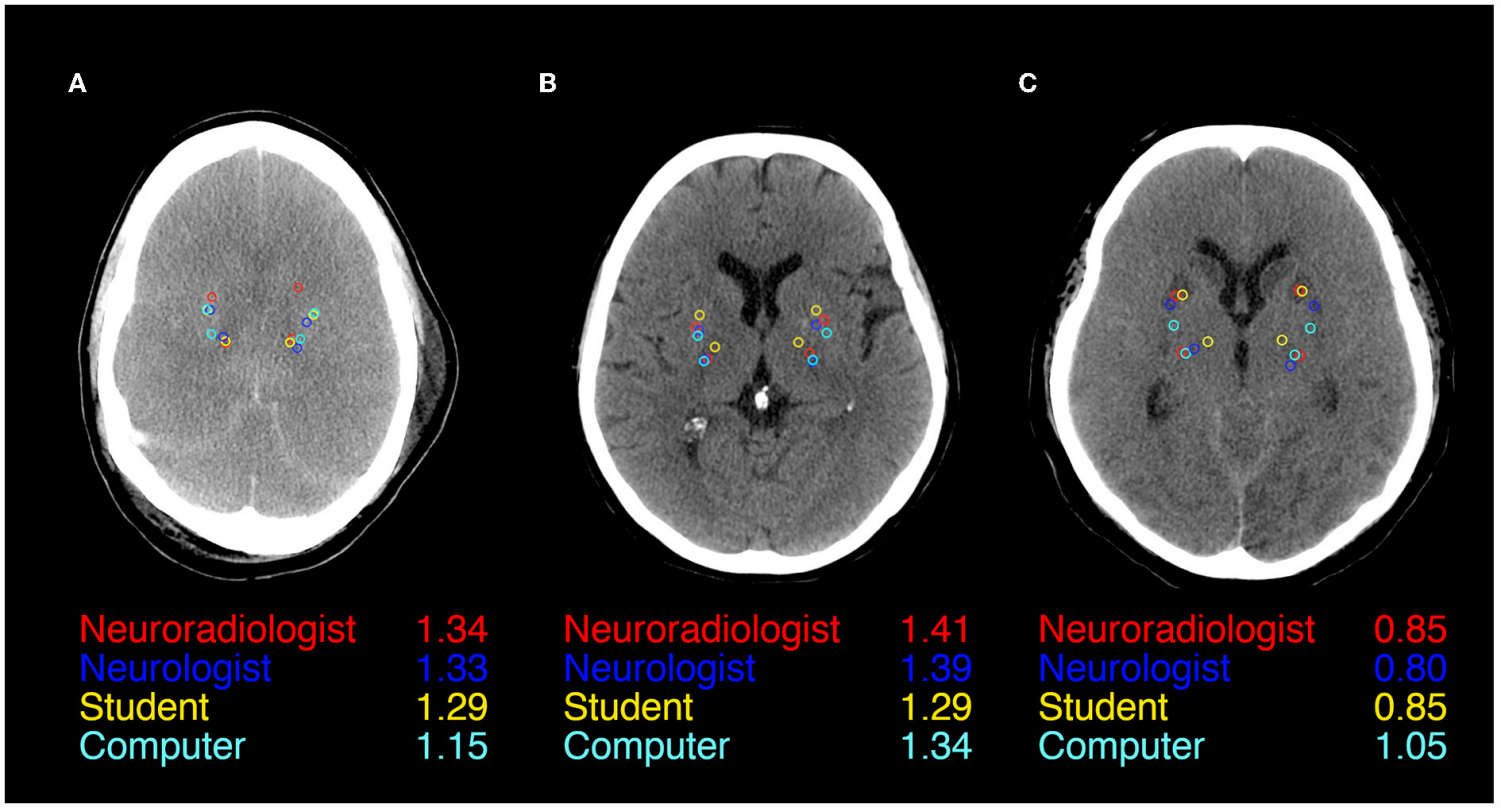
ROI Placement during GWR assessment in Putamen and Posterior Limb of the internal Capsule (PLIC) in CTs of three patients after cardiac arrest **(A–C)**. All four raters (Neuroradiologist in red, Neurologist in blue, Student in yellow, and Computer in cyan) and GWRs displayed.

**Figure 2:**
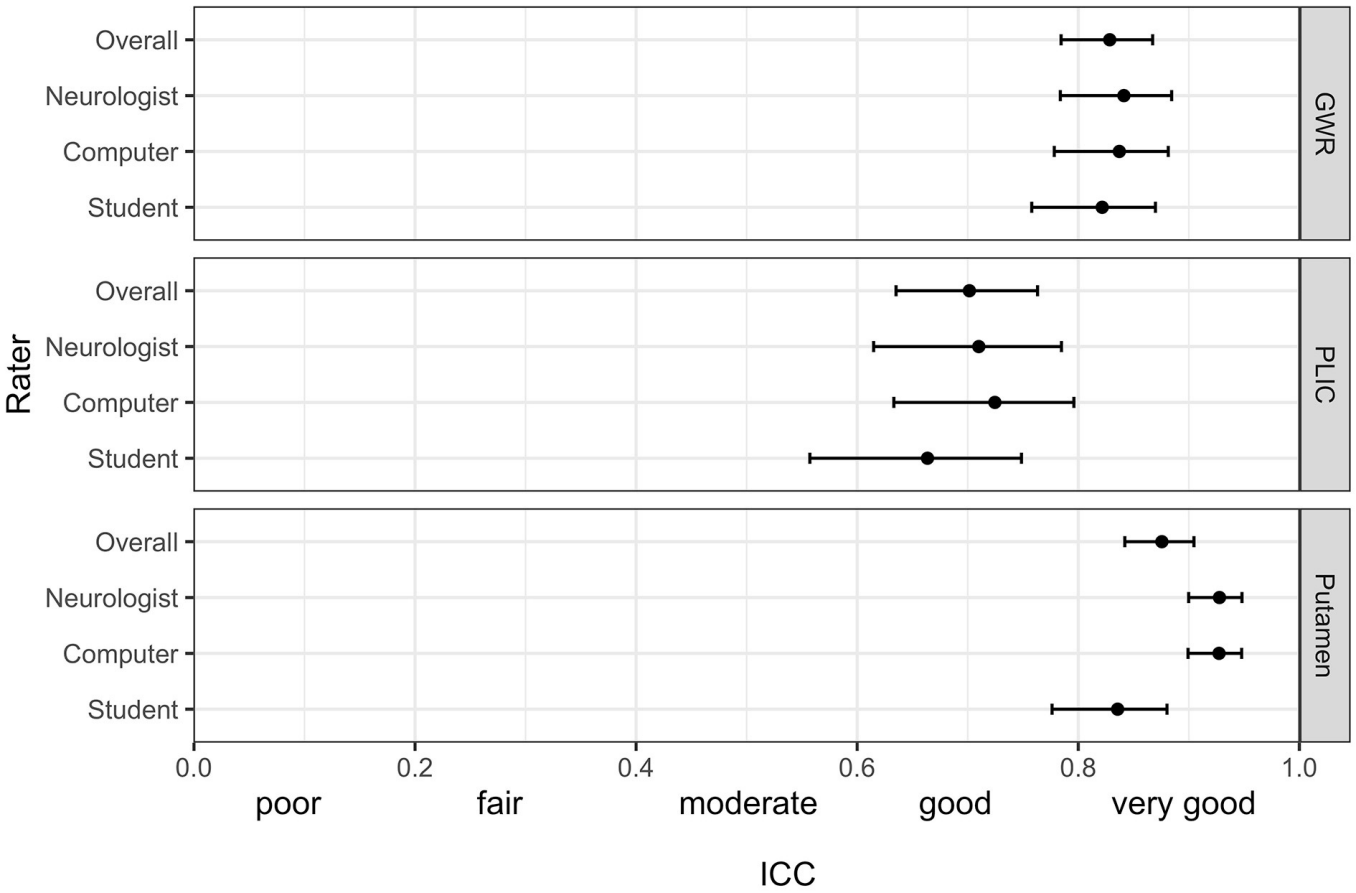
Intraclass correlation (ICC) (95% CI) for the pairwise agreement of three different raters with the study neuroradiologist in GWR and ROI assessment (Putamen and PLIC) in Hounsfield Units (HU) in CTs after cardiac arrest.

**Figure 3:**
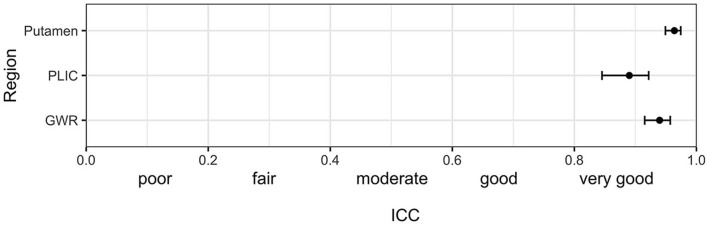
Intraclass correlation (ICC) (95%-CI) for the combined agreement of three human raters (mean of student, neurologist and neuroradiologist) with the computer algorithm in GWR and ROI assessment (Putamen and PLIC) in Hounsfield Units (HU) in CTs after cardiac arrest.

Figure 1 illustrates ROI placement in 3 patients with different extent of post hypoxic brain damage. For the measurement of putamen radiodensity, ICC for agreement with the study neuroradiologist (reference standard) was *very good* for all three raters (student 0.83, neurologist 0.92, computer 0.92). The agreement for PLIC was lower, although still at *good* levels (student 0.66, neurologist 0.71, computer 0.72; Figure 2). The student had the lowest correlation with the neuroradiologist in both regions (Putamen ICC 0.84, CI 0.78–0.88; PLIC 0.66 0.56–0.75) with differences ranging up to a maximum of 4.4 HU for PLIC. Moreover, the computers' agreement with the average of all human raters was *very good* (ICC Putamen 0.96, CI 0.95–0.97; PLIC 0.98, CI 0.84–0.92; Figure 3).

**Figure 4:**
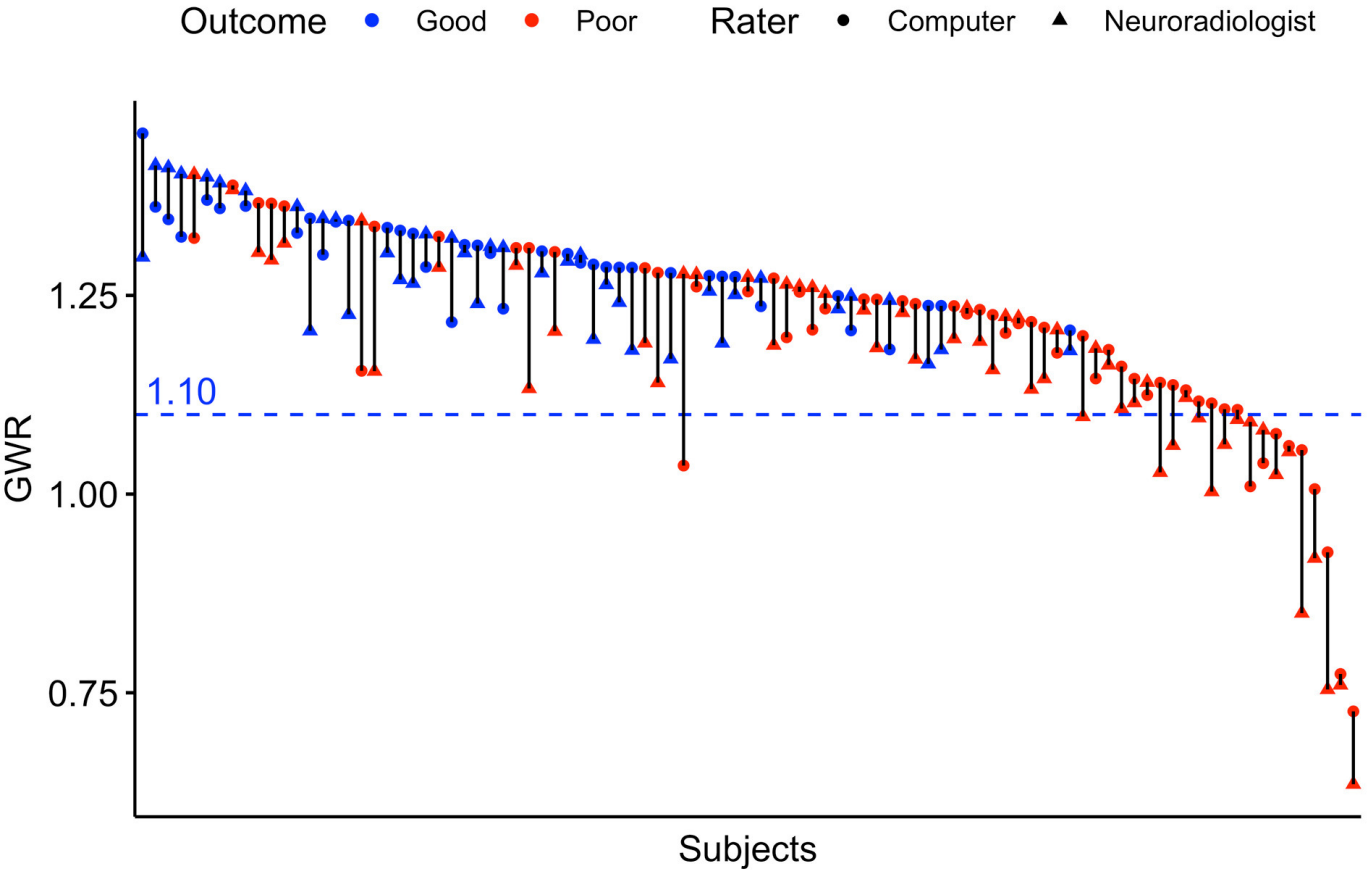
Pairwise GWR values for *n* = 95 patients rated by computer and neuroradiologist. Blue pairs representing patients with good outcome (CPC 1–3), red pairs poor outcome (CPC 4–5). Triangles for manual rater (neuroradiologist), dots for computer. Blue dotted line at GWR-cutoff = 1.10.

The raters' overall agreement with the study neuroradiologist on the GWR was *very good* (overall ICC 0.83; CIs 0.78–0.87), with 95%-CIs ranging into *good* agreement (Figure 2). Furthermore, the agreement between the computer and the average of all three human raters was *very good* (0.93; CI 0.91–0.96, Figure 3). However, when comparing individual GWR values between computer and neuroradiologist (ICC 0.84; CI 0.78–0.88), differences ranged from a minimum of 0.003 to a maximum of 0.24 with disagreement across the pre-defined cut-off for severe HIE (GWR < 1.10) in eight poor neurological outcome patients (Figure 4, CT image examples in Supplementary material). Similarly, the neurologist disagreed in six poor neurological outcome cases and the student nine cases with the neuroradiologist (Supplementary Figure 2). No disagreement with the neuroradiologist across the cutoff was observed in any good neurological outcome patient for any rater.

### Prognostic variability

**Figure 5:**
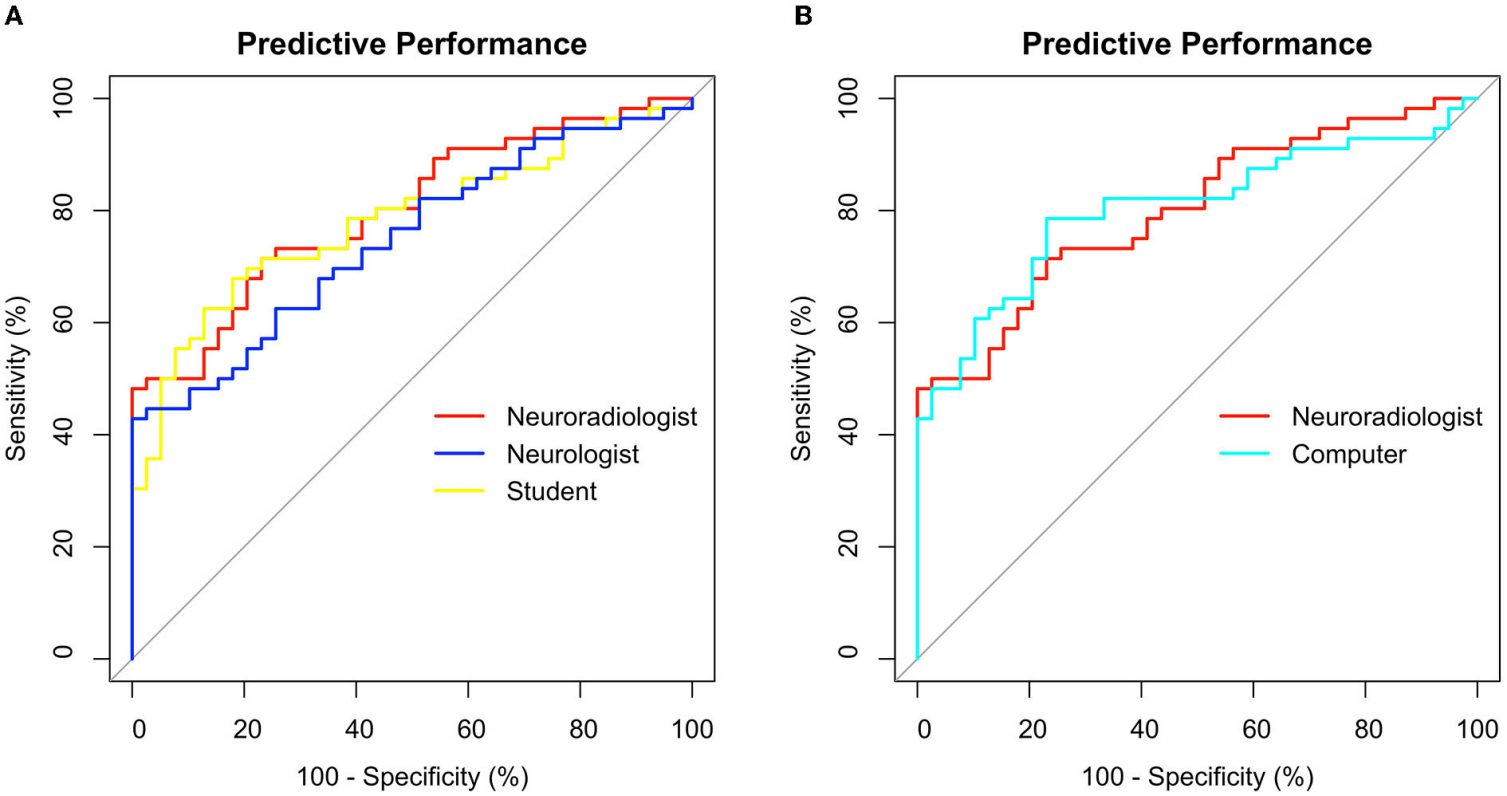
Receiver operating characteristics (ROC) for poor outcome prediction after CA using GWR rated by **(A)** 3 different human raters **(B)** Neuroradiologist vs. Computer. Sensitivity and Specificity in %.

Median GWRs were significantly lower in patients with poor neurological outcome than in patients with good neurological outcome, regardless of the rater (Supplementary Table 1). Predictive performance for poor neurological outcome prediction by GWR, quantified by the area under the ROC curve (AUC) was equally good for neuroradiologist and computer (AUC 0.80; CI 0.71–0.89) and lower for the neurologist (0.74; CI 0.65–0.84) and student (0.78; CI 0.69–0.87) (Figure 5). The differences in AUC between all raters were not statistically significant. Sensitivity at GWR < 1.10, a 100% specificity cutoff for all raters ranged between 18% for the computer and was highest at 29% for the neuroradiologist (Supplementary Table 2).

## Discussion

Our main findings are:

1. The Inter-rater agreement on the Gray-White Matter Ratio measured in head CTs after CA was very good between 3 human raters across different levels of expertise.
2. The Inter-rater agreement on GWR measured in head CTs was very good between human raters and a computer algorithm.
3. Inter-rater agreement was lower for ROIs placed in the posterior limb of the internal capsule than for those placed in the putamen.
4. Despite high overall agreement, considerable deviations in GWR between raters were observed in individual patients. In our cohort, at a GWR threshold of 1.10, this did not lead to any false poor neurological outcome predictions.

In this study investigating the Inter-rater variability of quantitative head CT assessment in patients after cardiac arrest, Inter-rater agreement was considerably better than in studies investigating descriptive, qualitative assessment (9, 10). Previous evidence on the subject had been inconclusive with one study reporting moderate agreement between three human raters and another study reporting good agreement between four human raters (23, 24). We provide the first data for the Inter-rater agreement between human raters and an atlas-based computer algorithm.

We observed the highest variability in the HU measurements of the PLIC-ROI, which mainly accounted for the variability of GWR values. ROI-placement in the Putamen was more consistent. This is illustrated in our visual examples and might be due to a less evident visual delineation of internal capsule in contrast to a clear structural border of the basal ganglia (Figure 1). Another reason could be the increasing difficulty with visually identifying anatomical regions when gray-white matter differentiation is lost due to brain edema (Figure 1A). We also provide evidence that this effect could partly be solved by automated delineation: we observed the highest agreement for the PLIC-ROI between the computer and the neuroradiologist. Considerable GWR deviations or disagreements across the cutoff for HIE occurred both in CTs with extensive as well as subtle pathological changes (Supplementary Figures 3, 4). Three main sources for variability can be observed in these images: First, raters seem to have a preference as to where to place ROIs within the target structures. Future studies with manual placement should therefore standardize ROI placement i.e., by defining the position within the anatomical region and the ROIs relation to other adjacent landmarks. Second, due to small structural focal hypo- or hyperdensitites (e.g., vessels and small lacunar defects) HU values can also vary if ROIs are placed correctly within the anatomic region. We therefore suggest that raters consider the HU value during ROI placement to be in an area representative of the regions radiodensity. Both problems can be solved if instead of circular ROIs, the whole anatomical region is delineated and its radiodensity averaged, an approach that has been successfully used in automated CT quantification by our group an others (14, 25, 26). Third, there are cases where ROIs are misplaced by the rater. Therefore, the results of manual and/or automated ROI placements should be visually re-inspected after qualitative and quantitative analysis are completed to identify cases of misplacement.

Although overall agreement on the GWR values as measure of the degree of edema was very good, we observed considerable variability in individual patients in both neurological outcome groups, the extent of which has not been previously reported. In some cases, this also affected whether a patient was above or below our predefined cut-off for “severe HIE” (GWR < 1.10). In our cohort this exclusively occurred in poor neurological outcome patients, no good neurological outcome patient was misclassified as below the cut-off. Thus, it influenced the sensitivity while retaining the high specificity for poor neurological outcome prediction. It is still possible, however, that the degree of variability observed in some patients of our study might occur in good neurological outcome patients in another cohort. We therefore advise caution for solely relying on the GWR values as prognostic information derived from CT after CA, especially when the values are close to the cut-off. We suggest that CT interpretation should be based on an SOP that integrates both qualitative and quantitative analysis before concluding on the absence or presence of HIE. This information should then always be put into the context of the results of other diagnostic modalities (EEG, SEP, and serum biomarkers).

Despite a tendency toward more consistent ROI placement in experienced human raters, we observed no significant difference in overall prognostic performance between all 4 raters, even though a clinically unexperienced graduate student who was pre-trained for the task was included. The results contrast with the considerable Inter-rater variability of experienced clinicians in qualitative assessment (9) and underscores that CT quantification reduces Inter-rater variability. We therefore recommend a protocol-based training for physicians doing GWR-assessment in future studies and in the clinical routine. Using a standardized automated method could eliminate the problem of Inter-rater variability overall by assisting the rating physician through visually delineating structures or recommending ROIs.

In this study, GWR performed similar in poor neurological outcome prediction as compared to previous studies by our group and others (13, 14, 22). We account the lower sensitivity of 18–28% in this study to the design that did not stratify between early CTs obtained within 24 h and late CTs performed later than 24 h after CA. The majority of CTs included in our study were early CTs. In our cohort, GWR < 1.10 was a safe and reliable cutoff for poor neurological outcome prediction at 100% specificity, regardless of the rater. Using a higher cutoff in our cohort improved sensitivity considerably for some raters without trade-offs in specificity, for instance up to 48% for the neuroradiologist or 42% for the computer (Figure 5) but would have further increased the risk of misclassification of patients when applying to another cohort (Supplementary Figure 2).

### Limitations

There are potential additional sources of rater-independent variability such as the type of CT scanner, acquisition parameters or post-processing software (27). We did not examine intra-rater agreement as additional source of variability. Because of the low number of raters, the three human raters' performance is not representative of that of their group (neuroradiologist, neurologist, and student). CT images were used as part of a multimodal approach to decide on continuation or withdrawal of life-sustaining therapy (WLST). Thus, we cannot exclude self-fulfilling prophecy (28). Prognostication was always based on careful consideration of multimodal diagnostics and a considerable observation period. Because neurological outcome was assessed at hospital discharge, we cannot exclude later improvement in patients assigned to the poor neurological outcome group. We therefore assigned CPC 3 (severe neurological deficit) to the good neurological outcome group to prevent overly pessimistic prognosis. As this was a single-center study, the subject should be further studied on larger, prospective cohorts in different clinical settings.

## Conclusion

Inter-rater agreement on quantitative head CT analysis after CA was very good in between human raters with different levels of expertise and a computer algorithm. As we observed considerable Inter-rater variability in a few individual patients, we advise caution for solely relying on GWR values as prognostic information derived from CT after CA. The results underscore the need for strategies to further standardize quantitative head CT analysis and for multimodal prognostication in general. Inter-rater variability should be investigated and considered in all future studies of CT quantification.

## Data availability statement

The datasets presented in this article are not readily available because of ethical and privacy restrictions. Requests to access the datasets should be directed to the corresponding author.

## Ethics statement

The studies involving human participants were reviewed and approved by Ethikkommission der Charité—Universitätsmedizin Berlin. Written informed consent for participation was not required for this study in accordance with the national legislation and the institutional requirements.

## Author contributions

MK undertook literature research, data collection, manual and automated image analysis, statistical analysis, and drafted the manuscript. ZC rated images and performed statistical analysis. MS rated images. CP, MS, CS, CG, and CL critically reviewed the manuscript. All authors contributed to the article and approved the submitted version.

## Funding

MK is participant in the BIH Charité Junior Digital Clinician Scientist Program funded by the Charité—Universitätsmedizin Berlin, and the Berlin Institute of Health at Charité (BIH). CL is participant in the BIH Clinical Fellow Program funded by the Charité—Universitätsmedizin Berlin, and the Berlin Institute of Health at Charité (BIH). Both received a grant from the Laerdal Foundation for research in post cardiac care.

## Conflict of interest

Author CL reported institutional fees for lecturing from BD Bard, Zoll, and Bristol Meyer Squibb outside the submitted work. Author CS reported receiving personal fees for consultancy from Philips, BD Bard, Zoll, Rhinocill, and Sedana Medical outside the submitted work. The remaining authors declare that the research was conducted in the absence of any commercial or financial relationships that could be construed as a potential conflict of interest.

## Publisher's note

All claims expressed in this article are solely those of the authors and do not necessarily represent those of their affiliated organizations, or those of the publisher, the editors and the reviewers. Any product that may be evaluated in this article, or claim that may be made by its manufacturer, is not guaranteed or endorsed by the publisher.
