# Supplementary material for: Inter-rater agreement between humans and computer in quantitative assessment of computed tomography after cardiac arrest
Source: Front Neurol. 2022 Oct 13;13:990208. doi: 10.3389/fneur.2022.990208 (PMC9606648; doi:10.3389/fneur.2022.990208)

Interrater Agreement between Humans and Computer in Quantitative Assessment of Computed Tomography after Cardiac Arrest

-

Supplementary Material

Martin Kenda, MD; Zhuo Cheng; Christopher Guettler, MD; Christian Storm, Prof., MD;

Christoph J. Ploner, Prof., MD; Christoph Leithner, MD; Michael Scheel, MD

Supplementary Table 1 – Median (IQR) Gray-White Matter Ratios of n=95 patients per rater and neurological outcome.

**Rater**	**Good Outcome (CPC 1-3)** *n=39*	**Poor Outcome (CPC 3-4)** *n=56*	**p-value MWU test**
Neuroradiologist	1.27 (1.24-1.32)	1.18 (1.10-1.25)	<0.01
Computer	1.30 (1.27-1.33)	1.21 (1.12-1.26)	<0.01
Neurologist	1.28 (1.24-1.34)	1.22 (1.12-1.28)	<0.01
Student	1.26 (1.22-1.29)	1.18 (1.10-1.25)	<0.01

Supplementary Table 2 – Receiver Operating Characteristics (ROC) for prognostication of poor neurological outcome by GWR. p-Value (DeLong's test) for AUC-comparison with the neuroradiologist.

**Rater**	**Area under the ROC curve** (95% CI)	**% Specificity** at GWR<1.10 (95% CI)	**% Sensitivity** at GWR<1.10 (95% CI)	**p-Value** (DeLong's test vs. Neuroradiologist)
Neuroradiologist	0.80 (0.71-0.89)	100 (88-100)	29 (18-41)	
Computer	0.80 (0.71-0.89)	100 (88-100)	18 (9-29)	0.9482
Neurologist	0.74 (0.65-0.84)	100 (88-100)	21 (11-32)	0.2434
Student	0.78 (0.69-0.87)	100 (88-100)	27 (16-39)	0.6822

Supplementary Figure 1 – Patient selection and exclusions

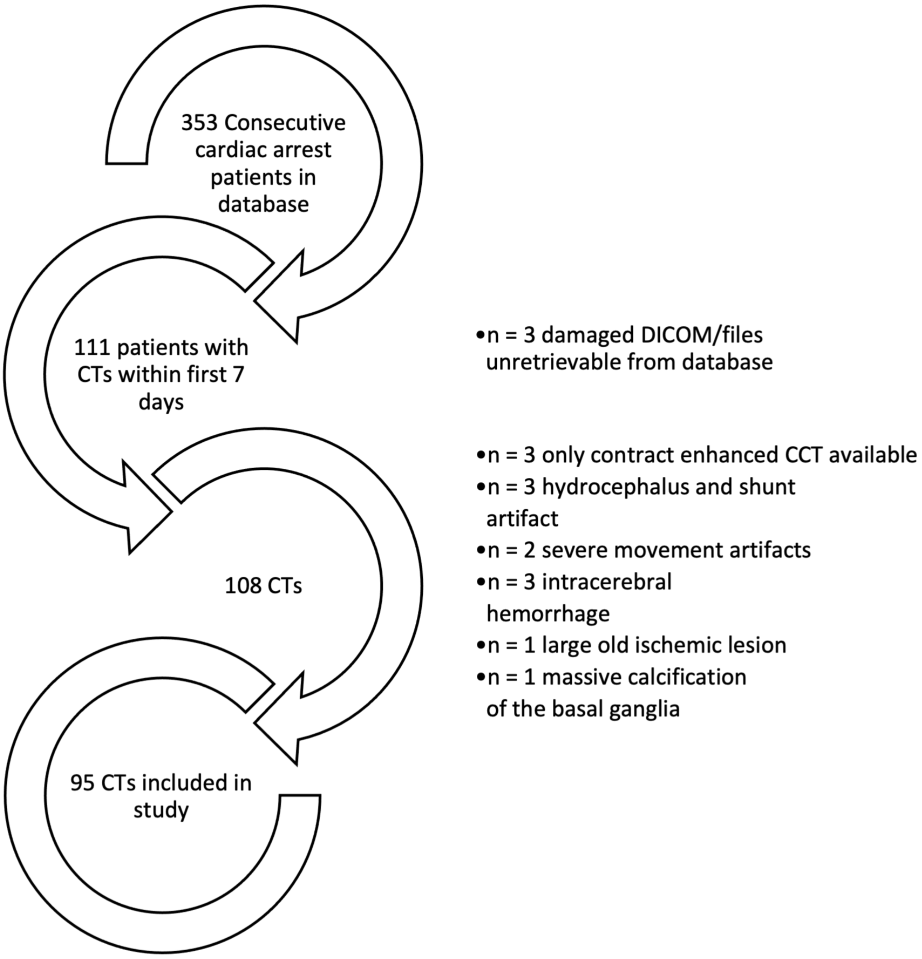

Supplementary Figure 2 - Pairwise GWR values for *n=95* patients rated by **(A)** neuroradiologist/neurologist, **(B)** neuroradiologist/student. Blue pairs representing patients with good neurological outcome (CPC 1-3), red pairs with poor neurological outcome (CPC 4-5). Dots for neuroradiologist, triangles for neurologist (A) and student (B). Blue dotted line at GWR-cutoff = 1.10.

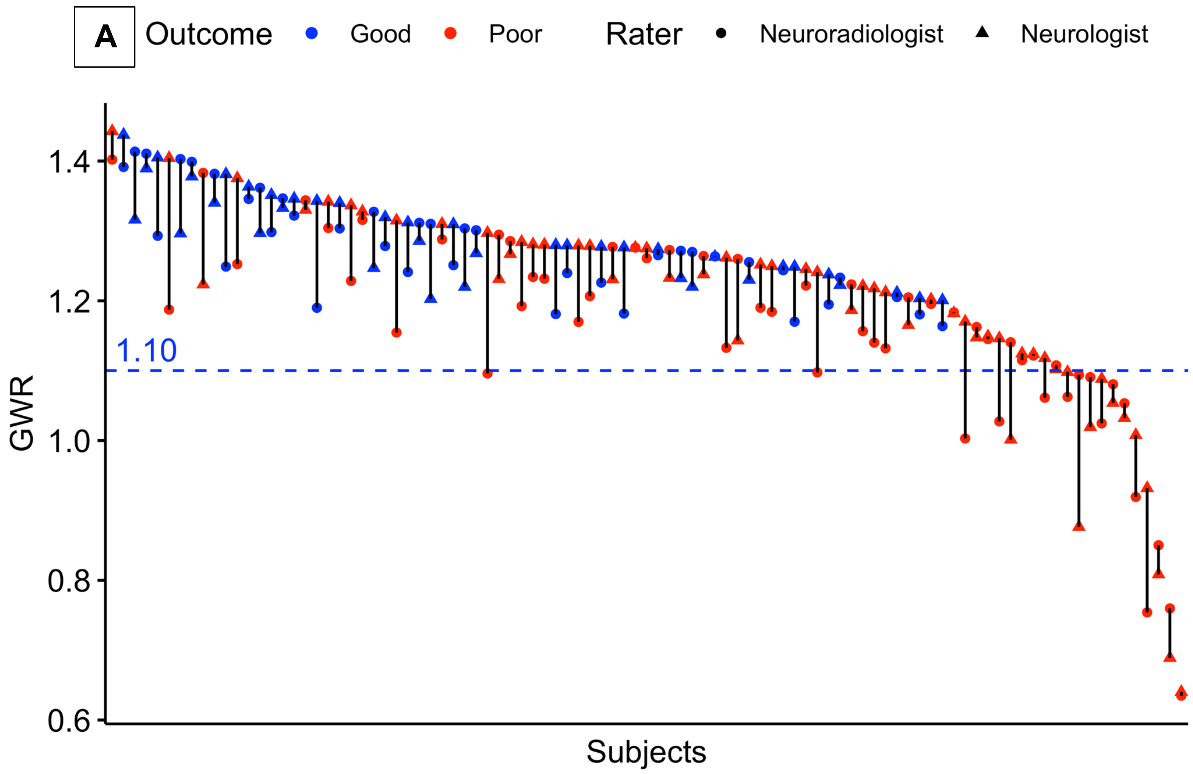

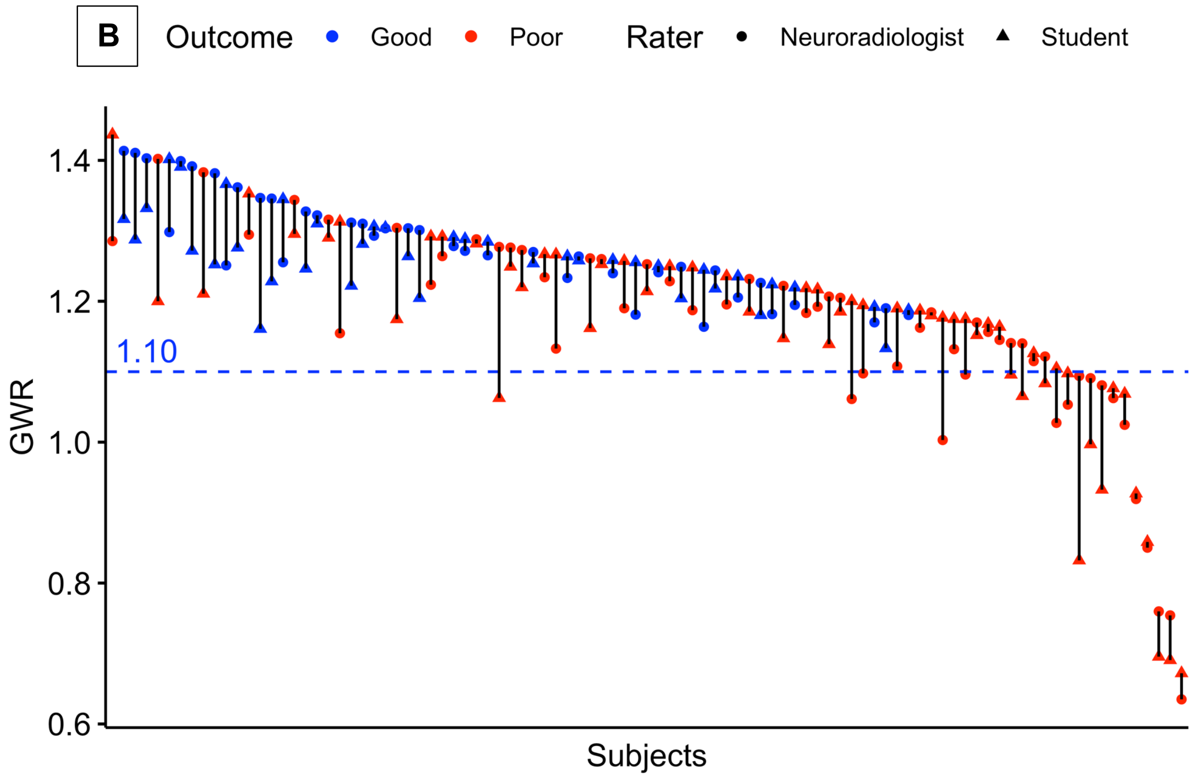

Supplementary Figure 3 – ROI Placement for GWR assessment in Putamen and Posterior Limb of the internal Capsule (PLIC) in CTs of five patients after cardiac arrest with the biggest GWR-difference between Neuroradiologist (red) and Computer (cyan).

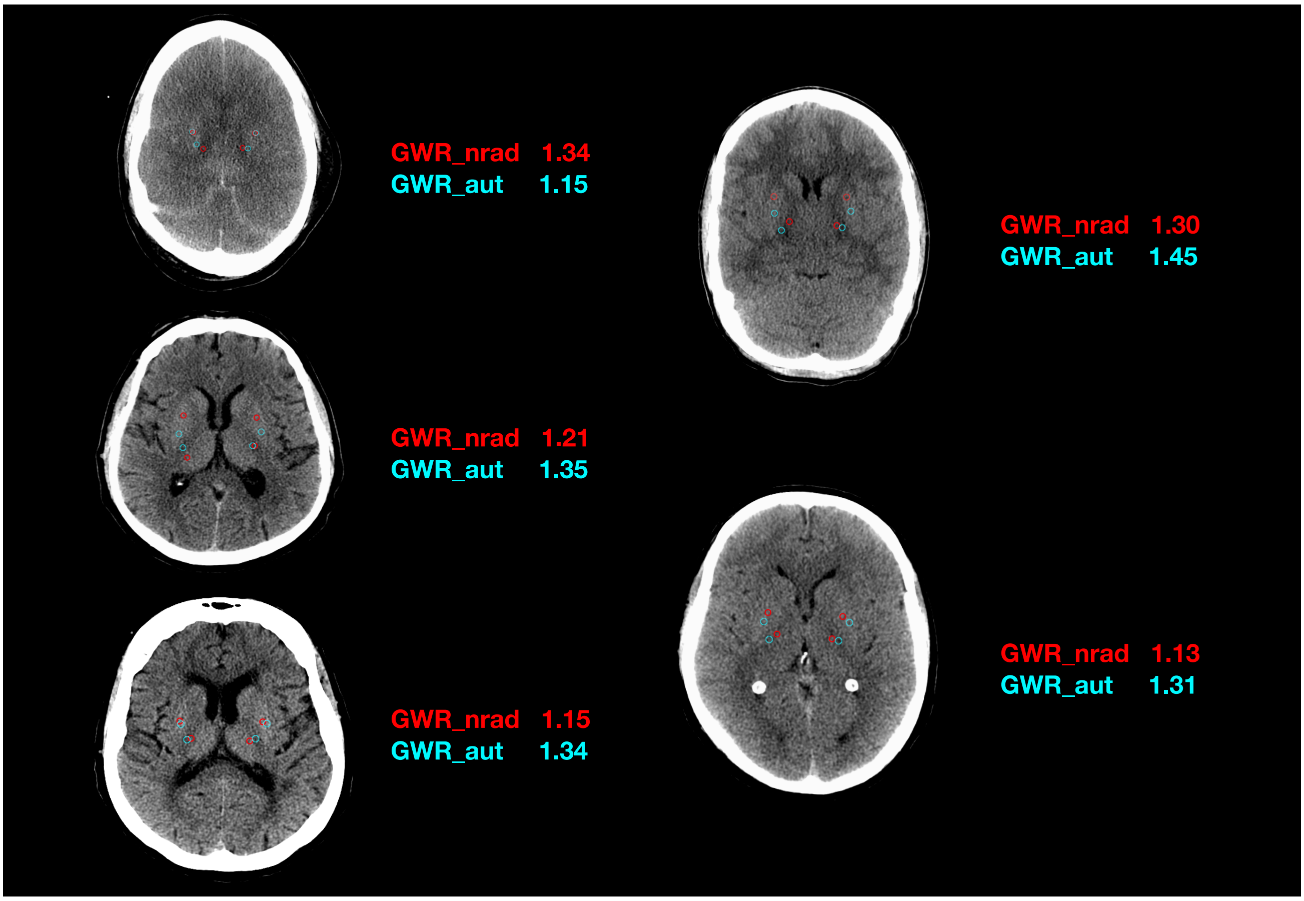

Supplementary Figure 4 – ROI Placement for GWR assessment in Putamen and Posterior Limb of the internal Capsule (PLIC) in CTs of five CA patients with disagreements on GWR with respect to the pre-defined cutoff for "severe HIE" (<1.10) between Neuroradiologist (red) and Computer (cyan).

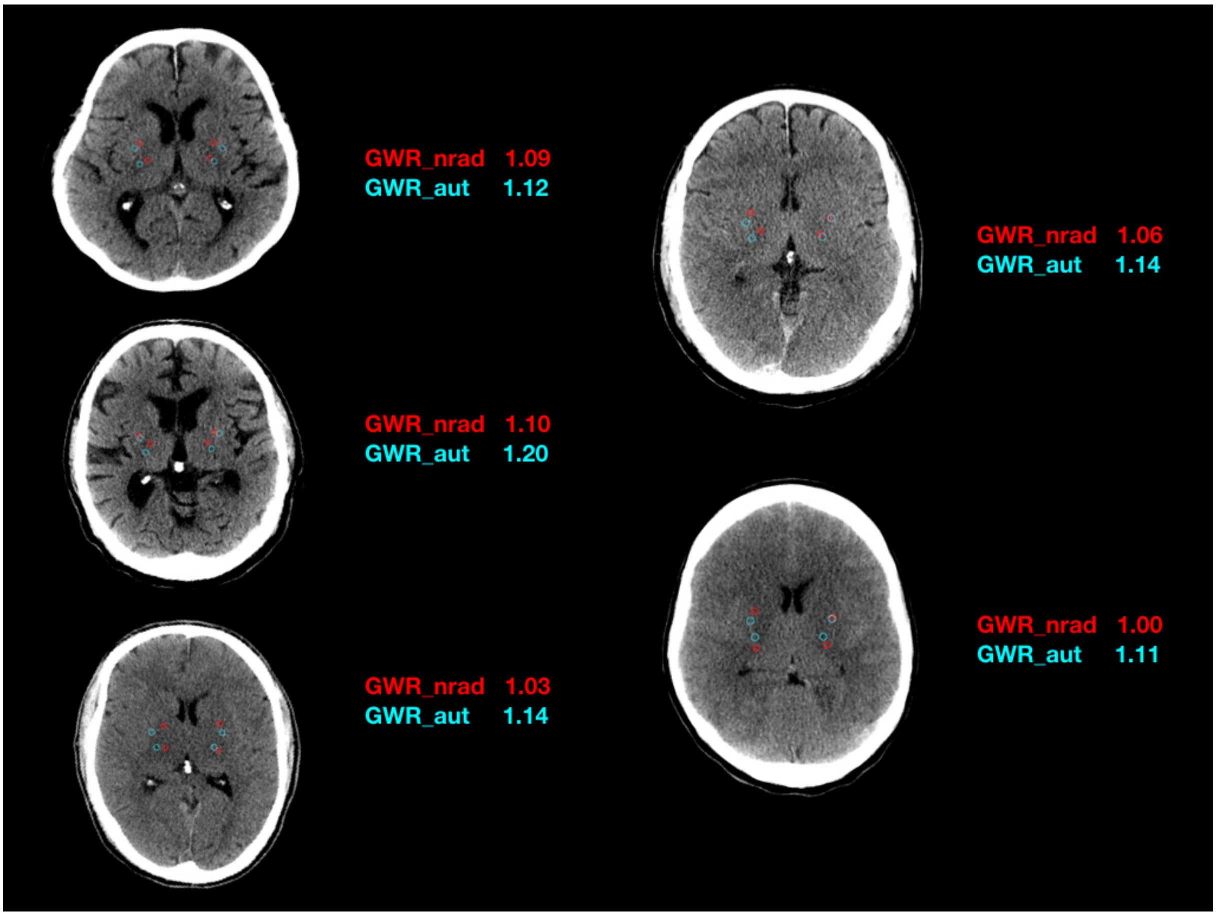

Supplement: Supplementary file 1 [file Data_Sheet_1.pdf]
